# Preoperative ovarian vascular parameters and perioperative factors associated with ovarian reserve marker decline after laparoscopic endometrioma cystectomy: a retrospective prediction study

**DOI:** 10.3389/fsurg.2026.1964292

**Published:** 2026-10-21

**Authors:** Mengmeng Wu, Mei Qin, Linrui Zhang

**Affiliations:** 1 Department of Ultrasound Medicine, Wenzhou People’s Hospital/Wenzhou Maternal and Child Health Care Hospital, Wenzhou, Zhejiang, China; 2 School of Nursing, Guangxi Health Science College, Nanning, Guangxi, China; 3 Health Management Centre, Yueyang People’s Hospital, Yueyang, Hunan, China

**Keywords:** doppler hemodynamics, laparoscopic cystectomy, ovarian endometrioma, ovarian reserve, prediction model, resistance index, three-dimensional ultrasound, vascularization index

## Abstract

**Background:**

Laparoscopic cystectomy is a commonly used surgical treatment for ovarian endometrioma, but postoperative reduction in ovarian reserve markers is a recognized concern. Preoperative identification of women at increased risk may support counseling and surgical planning. This study evaluated whether multidimensional ovarian vascular and hemodynamic ultrasound parameters are associated with subsequent decline in ovarian reserve markers.

**Objective:**

To evaluate the association of preoperative multidimensional ultrasound parameters with postoperative ovarian reserve marker decline and to develop an exploratory perioperative prediction model.

**Methods:**

A retrospective cohort of 280 reproductive-age women undergoing laparoscopic ovarian endometrioma cystectomy (May 2021-May 2024) was analyzed. Transvaginal color Doppler ultrasound on menstrual cycle days 2–5 yielded 2D morphological parameters [ovarian volume [OV], antral follicle count [AFC]], 3D vascular parameters [vascularization index [VI], flow index [FI], vascularization-flow index [VFI]], and spectral Doppler parameters (resistance index [RI], pulsatility index [PI], peak systolic velocity [PSV], end-diastolic velocity [EDV]). Serum AMH, FSH, LH, and E2 were measured. Ovarian reserve markers were reassessed at 3, 6, and 12 months. The primary 6-month outcome was a study-specific composite decline in ovarian reserve markers. Exploratory multivariable logistic regression was used to construct a perioperative model, and internal optimism was estimated by 1,000 bootstrap resamples. Apparent discrimination was assessed by ROC analysis.

**Results:**

At 6 months, 118 of 280 patients (42.1%) met the study-specific composite outcome. Women meeting the outcome had lower preoperative VI, VFI, FI, PSV, and EDV and higher RI and PI (all *P* < 0.01). Pearson correlations showed moderate associations of vascular parameters with 6-month AMH and FSH levels. AMH decreased after surgery in both groups, with a greater reduction in patients meeting the composite outcome; significant effects of time, group, and group-by-time interaction were observed (all *P* < 0.001). The exploratory perioperative logistic model retained age, bilateral cysts, preoperative maximum cyst diameter, intraoperative r-AFS score, preoperative VI, and preoperative RI. Higher VI was associated with lower odds of the composite outcome, whereas higher RI was associated with greater odds. The composite model had an apparent AUC of 0.888 (95% CI 0.845–0.923), which was higher than that of VI (AUC=0.702) or RI (AUC=0.699) alone. Bootstrap internal validation yielded a corrected AUC of 0.878. At a probability cutoff of >0.57, sensitivity and specificity were 76.3% and 90.1%, respectively, with a PPV of 84.9% and an NPV of 83.9%. Because r-AFS is intraoperative and no external validation was performed, this is a perioperative rather than a strictly preoperative model and requires external validation before clinical application.

**Conclusions:**

Preoperative ovarian vascular and hemodynamic ultrasound parameters, particularly VI and RI, were associated with postoperative decline in ovarian reserve markers. The composite model showed good apparent discrimination and modest optimism on internal bootstrap validation; however, external validation and comparison with models incorporating established baseline ovarian reserve markers are required before clinical implementation.

## 1 Introduction

Ovarian endometrioma (OE) is a common manifestation of endometriosis and may adversely affect reproductive potential and ovarian reserve (1). Systematic evidence indicates that endometrioma cystectomy can be followed by a measurable decline in serum anti-Müllerian hormone (AMH), although the magnitude and persistence of this decline vary among studies (2). Current ESHRE guidance emphasizes individualized management according to symptoms, reproductive goals, ovarian reserve, previous surgery, and cyst characteristics rather than routine surgery for every endometrioma (3). Independent of surgery, women with endometrioma may already have reduced ovarian reserve compared with unaffected women, which complicates interpretation of postoperative changes (4). A separate meta-analysis has likewise shown short-, medium-, and long-term reductions in AMH after laparoscopic endometrioma surgery (5).

Laparoscopic cystectomy is therefore a commonly used, but context-dependent, surgical approach for selected patients with OE (6). Comparative evidence suggests that cystectomy and ablative techniques may have different effects on postoperative ovarian reserve (7), and the method used to achieve hemostasis may also influence AMH and AFC preservation (8). Clinical studies have explored tissue-sparing hemostatic approaches after cystectomy (9), while CO2 laser vaporization has been investigated as an alternative technique intended to reduce ovarian tissue injury (10).

AMH and antral follicle count (AFC) are established markers used to assess ovarian reserve, although they should be interpreted in the appropriate clinical context (11). Meta-analytic evidence supports the usefulness of both AMH and AFC for characterizing ovarian response and reserve-related status (12). Beyond these conventional markers, Doppler ultrasonography can characterize ovarian vascular flow and resistance (13). Three-dimensional power Doppler provides vascularization index (VI), flow index (FI), and vascularization-flow index (VFI), while spectral Doppler provides resistance index (RI), pulsatility index (PI), peak systolic velocity (PSV), and end-diastolic velocity (EDV). A meta-analysis has reported associations between ovarian Doppler indices and markers of ovarian dysfunction (14).

Several factors may influence postoperative ovarian reserve, including age, bilateral disease, baseline reserve, cyst burden, and surgical characteristics. Previous work has specifically examined predictors of ovarian reserve reduction after endometriotic cystectomy and emphasized the implications for fertility-preservation counseling (15). Age-related ovarian decline provides an additional biological context for interpreting postoperative reserve changes (16). More recent appraisal of systematic reviews further supports AMH as a particularly sensitive marker of ovarian reserve change after endometriotic cystectomy (17). However, the incremental predictive value of vascular ultrasound beyond established preoperative clinical variables, baseline AMH, and AFC remains uncertain.

Accordingly, this retrospective study evaluated whether preoperative ovarian vascular and hemodynamic ultrasound parameters were associated with postoperative decline in ovarian reserve markers after laparoscopic endometrioma cystectomy. A secondary aim was to construct an exploratory composite model and assess its apparent discrimination and internal optimism, while recognizing the need for external validation and comparison with models based on established preoperative ovarian reserve markers.

## 2 Materials and methods

### 2.1 Study design and participants

This single-center retrospective cohort included 280 reproductive-age women who underwent laparoscopic ovarian endometrioma cystectomy between May 2021 and May 2024 (Figure 1). The source population comprised women treated surgically for ovarian endometrioma during the study period. Surgical treatment was determined clinically rather than assigned by the study; recognized indications may include symptoms, infertility-related management, persistent or enlarging cysts, cyst characteristics, and individualized concern regarding diagnosis, consistent with contemporary endometriosis guidance (3). Because a standardized indication variable and the complete denominator of all women with ovarian endometrioma evaluated during the study period were not available in the retrospective dataset, indication-specific frequencies and the full screened denominator could not be reconstructed. This limits assessment of selection bias. The study was approved by the institutional ethics committee. The study was conducted in accordance with the Declaration of Helsinki, and the requirement for informed consent was handled in accordance with the approval of the institutional ethics committee (KY-2024-137).

**Figure 1:**
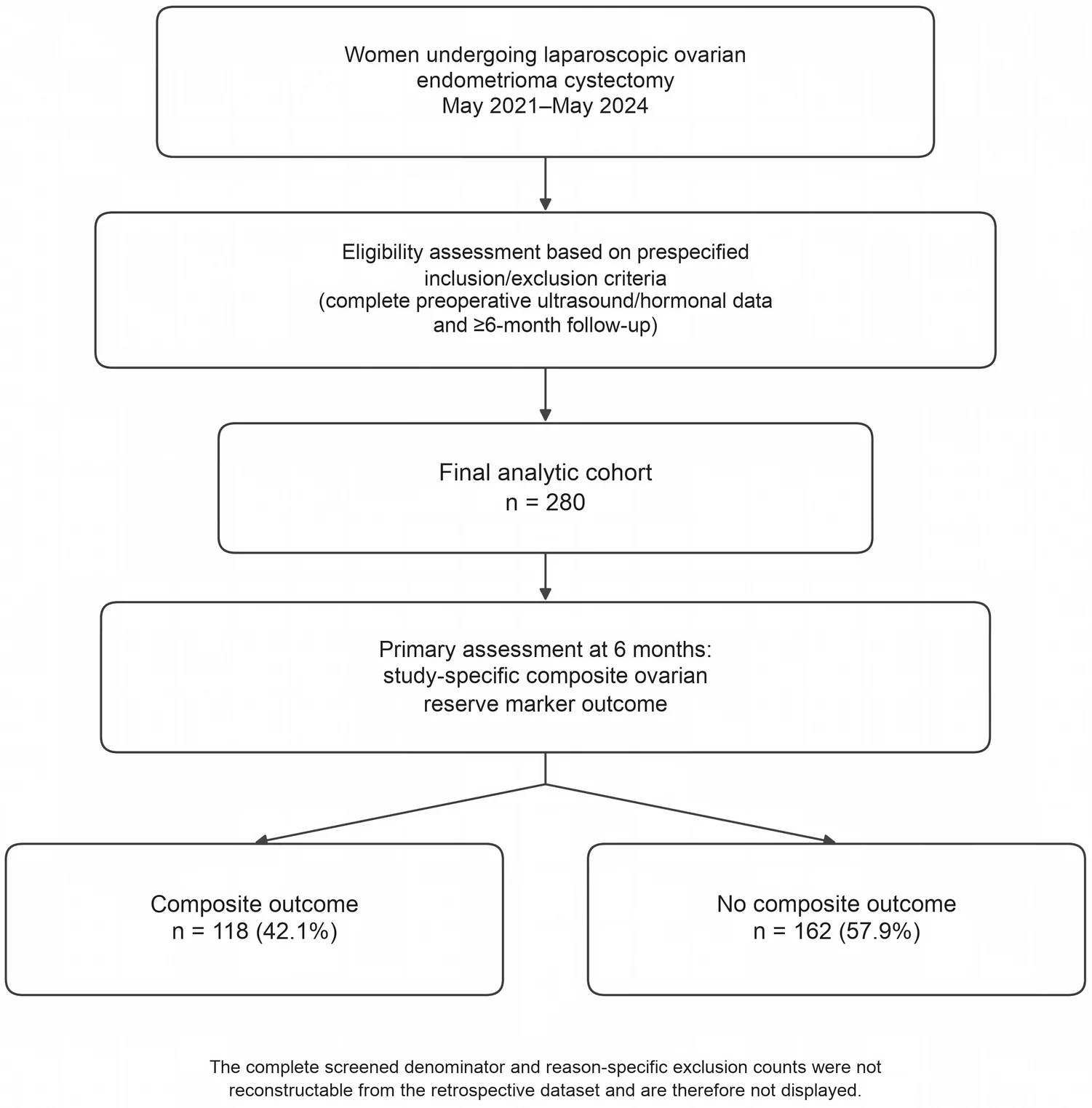
Patient recruitment pathway and postoperative classification according to the study-specific composite definition of ovarian reserve marker decline. A total of 280 women were included in the final analytic cohort; 118 (42.1%) met the composite outcome at 6 months and 162 (57.9%) did not. The complete screened denominator and reason-specific exclusion counts were not reconstructable from the retrospective dataset and are therefore not displayed.

Inclusion criteria were: (1) age 18–45 years; (2) preoperative diagnosis of ovarian endometrioma by transvaginal ultrasound and pelvic MRI; (3) postoperative histopathological confirmation; (4) regular menstrual cycles (21–35 days); (5) no hormonal medication within 3 months preoperatively; (6) complete preoperative ultrasound and hormonal data and postoperative ovarian-reserve assessments required for the primary analysis; and (7) at least 6 months of follow-up.

Exclusion criteria were coexisting ovarian tumors, previous ovarian surgery, polycystic ovary syndrome, premature ovarian insufficiency or other major endocrine disorders, concurrent assisted reproductive treatment, severe systemic organ dysfunction, pregnancy or lactation, postoperative malignant histology, loss to follow-up before the primary 6-month assessment, or incomplete records. The analytic cohort therefore represents patients with sufficiently complete preoperative Doppler and follow-up data. The retrospective dataset did not permit reconstruction of a complete reason-specific flow from all initially evaluated patients; this is acknowledged as a potential source of selection bias.

### 2.2 Preoperative multi-dimensional ultrasound assessment

All included participants underwent transvaginal color Doppler ultrasonography on menstrual-cycle days 2–5 using a GE Voluson E10 ultrasound system equipped with a 5–9 MHz transvaginal probe. Patients were examined in the lithotomy position after emptying the bladder. Conventional two-dimensional scanning was first performed to identify the ovaries and endometrioma lesions. Two-dimensional morphological measurements included ovarian volume (OV, cm^3^), calculated using the ellipsoid formula (length   ×   width   ×   height   ×   0.523), antral follicle count (AFC; follicles 2–10 mm), maximum cyst diameter, cyst volume, and ovarian stromal characteristics.

Three-dimensional power Doppler imaging was subsequently performed using a standardized acquisition protocol. The power Doppler gain was set at approximately 20–25 dB, with minor adjustment below the level at which background noise appeared. The pulse-repetition frequency was set at 0.6 kHz, and a low wall-motion filter was used to preserve low-velocity ovarian blood-flow signals. The transmitted power was maintained at a low-to-moderate level, and identical preset settings were used for all included examinations whenever technically feasible. The three-dimensional volume angle was set at 85°, and the acquisition sweep was performed with the probe held as steadily as possible to minimize motion artifact.

Three-dimensional datasets were analyzed using the VOCAL (Virtual Organ Computer-aided Analysis) technique. Manual contouring was performed at 30° rotational intervals, resulting in six contour planes for each ovarian volume. The region of interest was defined as the ovarian stroma surrounding the endometrioma. The cyst cavity itself was excluded from the vascular region of interest to avoid inclusion of nonvascular cystic content. Vascularization index (VI, %) represented the proportion of color-coded vascular voxels within the defined ovarian volume, flow index (FI) reflected the mean intensity of the detected Doppler signal, and vascularization-flow index (VFI, %) incorporated information on both vascularization and flow-signal intensity.

Spectral Doppler measurements were obtained from visible ovarian stromal arterial branches adjacent to, but not within, the cyst wall. A 2-mm sample volume was positioned within the vessel lumen. The insonation angle was maintained at ≤60° whenever angle correction was required. Peak systolic velocity (PSV, cm/s), end-diastolic velocity (EDV, cm/s), resistance index (RI), and pulsatility index (PI) were recorded. RI was calculated as (PSV−EDV)/PSV. Measurements were obtained after visualization of at least three consecutive stable waveforms, and the mean of three measurements was used for analysis.

For unilateral endometrioma, measurements from the affected ovary were used for the primary analysis. For bilateral endometriomas, the same parameters were measured in both ovaries and averaged to generate a participant-level value. The same aggregation principle was applied to VI, FI, VFI, RI, PI, PSV, EDV, OV, and AFC where applicable.

All examinations were performed by the same senior sonographer with more than 10 years of experience in gynecologic ultrasonography. Intraobserver reproducibility was evaluated in 20 participants by repeating the offline measurements after an interval of approximately 2 weeks. During the repeat assessment, the sonographer was blinded to the initial measurement results. Intraobserver agreement for VI, FI, VFI, PSV, EDV, RI, and PI was quantified using intraclass correlation coefficients (ICCs) with 95% confidence intervals, calculated using a two-way mixed-effects model for absolute agreement based on single measurements. Parameter-specific ICCs and corresponding 95% confidence intervals are presented in Supplementary Table S1. Because all ultrasound examinations and repeated measurements were performed by the same sonographer, interobserver reproducibility could not be assessed and is acknowledged as a limitation of the present study.

### 2.3 Serum hormonal assessment

Fasting venous blood samples were obtained on menstrual-cycle days 2–5 preoperatively and at 3, 6, and 12 months after surgery. Serum anti-Müllerian hormone (AMH), follicle-stimulating hormone (FSH), luteinizing hormone (LH), and estradiol (E2) were measured using chemiluminescent immunoassays on a Roche Cobas e602 platform. The laboratory reference intervals were 2.5–10.2 mIU/mL for FSH, 1.9–12.5 mIU/mL for LH, 19–144 pg/mL for E2, and 1.2–4.5 ng/mL for AMH. Baseline concentrations of these reproductive/endocrine biomarkers were summarized according to the 6-month composite outcome. Because AMH was the principal longitudinal marker of ovarian reserve in the present analysis, postoperative trajectories at 3, 6, and 12 months were analyzed primarily for AMH.

### 2.4 Surgical standardization

All procedures were performed by the same surgical team using standard three-port laparoscopic technique under general anesthesia (CO₂ pneumoperitoneum 12–15 mmHg). Complete cyst enucleation along the capsule plane was performed, with meticulous bipolar electrocoagulation for hemostasis and maximal preservation of normal ovarian cortex. Intraoperative data recorded included cyst laterality, maximum diameter, operative time, estimated blood loss, electrocoagulation duration, and adhesion severity (r-AFS score).

### 2.5 Definition of postoperative decline in ovarian reserve markers

The primary 6-month outcome was a study-specific composite endpoint defined as meeting at least one of the following criteria: (1) an AMH decrease of ≥25% from baseline; (2) an AFC decrease of ≥25% from baseline; (3) FSH ≥12 mIU/mL or an increase of ≥50% from baseline; or (4) postoperative oligomenorrhea, defined as a menstrual cycle >35 days, or amenorrhea. AMH and AFC are established ovarian reserve markers; however, no single international guideline was identified that validates this exact combination of components and thresholds as a unified definition of diminished ovarian reserve. The endpoint was therefore regarded as an operational study-specific definition of postoperative decline in ovarian reserve markers rather than as a universally accepted clinical diagnosis of diminished ovarian reserve. The frequency and overlap of the individual components were additionally analyzed to facilitate interpretation of this composite outcome (18).

### 2.6 Statistical analysis

Analyses were performed using SPSS 26.0. Continuous variables were summarized as mean ± SD and categorical variables as *n* (%). Distributional assumptions for continuous variables were assessed before between-group comparisons. Approximately normally distributed variables were compared using independent-samples t tests, whereas categorical variables were compared using the chi-square test or Fisher exact test, as appropriate. All statistical tests were two-sided, and *P* < 0.05 was considered statistically significant.

Pearson correlation analysis was used to evaluate associations between preoperative ovarian vascular/hemodynamic parameters and serum AMH and FSH concentrations at 6 months. Correlation coefficients were interpreted as unadjusted associations rather than evidence of causal relationships.

Longitudinal AMH changes were evaluated using a mixed-effects model. Time, outcome group, and the group-by-time interaction were entered as fixed effects, and participant was included as a random intercept to account for within-subject correlation. Complete AMH measurements were available for all 280 participants at baseline and at 3, 6, and 12 months after surgery; therefore, no longitudinal AMH observations were missing at the prespecified study time points.

Candidate predictors for the multivariable analysis were considered on the basis of clinical relevance, univariable associations, and potential collinearity. Because VI, FI, and VFI represent related 3D power-Doppler characteristics, they were not entered simultaneously into the primary model. VI was selected as the representative 3D vascularization parameter because it directly reflects the proportion of vascularized tissue within the defined ovarian volume, whereas RI was selected as the representative spectral Doppler resistance parameter. Age, bilateral cysts, preoperative maximum cyst diameter, intraoperative r-AFS score, VI, and RI were therefore included in the primary exploratory multivariable logistic regression model.

To examine whether the model findings depended on the choice of the 3D vascularization parameter, a sensitivity analysis was performed by replacing VI with VFI while retaining the remaining covariates unchanged. Odds ratios (ORs) and 95% confidence intervals (CIs) were calculated from the regression coefficients. For clinical interpretability, the RI effect was expressed per 0.1-unit increase. Multicollinearity was assessed using variance inflation factors.

Predicted probabilities from the final multivariable model were used to generate receiver operating characteristic (ROC) curves. Discrimination was quantified using the area under the ROC curve (AUC) with 95% CI. The optimal probability cutoff was selected by maximizing the Youden index. Sensitivity, specificity, positive predictive value (PPV), and negative predictive value (NPV) were calculated at this threshold. VI and RI were also evaluated individually by ROC analysis.

Internal validation was performed using 1,000 bootstrap resamples. Optimism was estimated as the mean difference between performance in each bootstrap sample and performance when the fitted model was evaluated in the original cohort. The optimism-corrected AUC was calculated by subtracting the estimated optimism from the apparent AUC. Bootstrap validation was interpreted as internal validation only and not as evidence of external transportability. Model calibration was assessed using the Hosmer–Lemeshow goodness-of-fit test, while recognizing that this test alone does not fully characterize calibration. Reporting was aligned with STROBE and TRIPOD principles.

## 3 Results

### 3.1 Clinical, perioperative, and baseline hormonal characteristics

A total of 280 women were included in the final analysis. At 6 months after laparoscopic endometrioma cystectomy, 118 patients (42.1%) met the study-specific composite definition of postoperative ovarian reserve marker decline, whereas 162 patients (57.9%) did not.

Compared with patients without the composite outcome, those who met the outcome were older (32.6 ± 5.0 vs. 29.3 ± 4.1 years, *P* < 0.001) and had a higher proportion of bilateral endometriomas (43.2% vs. 23.5%, *P* < 0.001). They also had a larger maximum cyst diameter and a higher r-AFS score. Operative time and estimated blood loss were modestly but significantly greater in the composite-outcome group, whereas BMI and menstrual-cycle length did not differ significantly between the groups (Table 1).

**Table 1:** Clinical and perioperative characteristics according to the 6-month composite ovarian reserve marker outcome.

Characteristic	Composite outcome (*n* = 118)	No composite outcome (*n* = 162)	t/*χ*^2^	*P* value
Age (years)	32.6 ± 5.0	29.3 ± 4.1	5.74	<0.001
BMI (kg/m^2^)	22.6 ± 2.5	22.3 ± 2.4	1.02	0.309
Menstrual-cycle length (days)	28.3 ± 2.1	28.1 ± 2.0	0.79	0.430
Bilateral cysts, *n* (%)	51 (43.2)	38 (23.5)	12.30	<0.001
Maximum cyst diameter (cm)	6.25 ± 1.48	5.44 ± 1.63	4.31	<0.001
r-AFS score	36.0 ± 11.0	26.2 ± 11.4	7.23	<0.001
Operative time (min)	65.9 ± 13.5	62.1 ± 13.9	2.26	0.025
Estimated blood loss (mL)	41.4 ± 15.3	37.6 ± 15.7	2.04	0.042

Data are presented as mean ± SD or *n* (%). BMI, body mass index; r-AFS, revised American Fertility Society score.

Baseline reproductive hormone profiles were also compared between the two groups. Patients who subsequently met the composite outcome had lower baseline AMH than those without the outcome (2.43 ± 0.86 vs. 2.76 ± 0.89 ng/mL, *P* = 0.002). In contrast, baseline FSH, LH, and E2 concentrations were comparable between groups (all *P* > 0.05) (Table 2). These findings indicate that the composite-outcome group had a modestly lower baseline ovarian reserve as reflected by AMH, whereas the remaining baseline reproductive hormone concentrations were broadly similar.

**Table 2:** Baseline reproductive hormone levels according to the 6-month composite ovarian reserve marker outcome.

Parameter	Composite outcome (*n* = 118)	No composite outcome (*n* = 162)	t	*P* value
AMH (ng/mL)	2.43 ± 0.86	2.76 ± 0.89	−3.11	0.002
FSH (mIU/mL)	6.42 ± 1.34	6.27 ± 1.25	0.95	0.341
LH (mIU/mL)	5.72 ± 1.68	5.59 ± 1.61	0.65	0.515
E2 (pg/mL)	51.6 ± 17.9	50.2 ± 18.4	0.64	0.523

Data are presented as mean ± SD. AMH, anti-Müllerian hormone; FSH, follicle-stimulating hormone; LH, luteinizing hormone; E2, estradiol.

Among the 118 patients who met the composite outcome, 104 (88.1%) had an AMH decline of ≥25% from baseline, 69 (58.5%) had an AFC decline of ≥25%, 37 (31.4%) met the FSH criterion, and 22 (18.6%) developed oligomenorrhea or amenorrhea. Because individual patients could meet more than one criterion, these components were not mutually exclusive. Forty-one patients (34.7%) met one criterion only, 47 (39.8%) met two criteria, 23 (19.5%) met three criteria, and 7 (5.9%) met all four criteria. The detailed distribution and overlap of the individual components are presented in Supplementary Table S2.

### 3.2 Preoperative ovarian morphological, vascular, and hemodynamic parameters

Patients who subsequently met the composite outcome had lower preoperative ovarian volume and AFC than those without the outcome. Significant differences were also observed in 3D power Doppler parameters, with lower VI, FI, and VFI in the composite-outcome group (Table 3).

**Table 3:** Comparison of preoperative ovarian morphological and 3D power Doppler parameters between outcome groups.

Parameter	Composite outcome (*n* = 118)	No composite outcome (*n* = 162)	t	*P* value
Ovarian volume, OV (cm^3^)	6.66 ± 2.02	8.01 ± 1.97	5.60	<0.001
Antral follicle count, AFC (n)	10.62 ± 2.80	12.15 ± 2.63	4.64	<0.001
Vascularization index, VI (%)	7.18 ± 2.30	8.87 ± 2.34	6.03	<0.001
Flow index, FI	34.97 ± 7.24	38.79 ± 6.62	4.52	<0.001
Vascularization-flow index, VFI (%)	2.49 ± 0.95	3.44 ± 1.09	7.73	<0.001

Data are presented as mean ± SD. OV, ovarian volume; AFC, antral follicle count; VI, vascularization index; FI, flow index; VFI, vascularization-flow index.

Spectral Doppler measurements showed lower PSV and EDV and higher RI and PI among patients who subsequently experienced ovarian reserve marker decline (Table 4). Together, these findings indicate that the two groups differed in several preoperative ovarian morphological and vascular/hemodynamic characteristics.

**Table 4:** Comparison of preoperative ovarian stromal artery spectral Doppler parameters between outcome groups.

Parameter	Composite outcome (*n* = 118)	No composite outcome (*n* = 162)	t	*P* value
Peak systolic velocity, PSV (cm/s)	11.19 ± 2.42	12.10 ± 2.43	3.08	0.002
End-diastolic velocity, EDV (cm/s)	4.19 ± 1.24	5.14 ± 1.29	6.22	<0.001
Resistance index, RI	0.626 ± 0.072	0.576 ± 0.064	6.00	<0.001
Pulsatility index, PI	1.35 ± 0.25	1.15 ± 0.26	6.45	<0.001

Data are presented as mean ± SD. PSV, peak systolic velocity; EDV, end-diastolic velocity; RI, resistance index; PI, pulsatility index.

### 3.3 Associations between preoperative vascular parameters and postoperative hormonal biomarkers

Pearson correlation analysis demonstrated significant associations between preoperative ovarian vascular characteristics and ovarian reserve-related hormonal levels at 6 months after surgery. Higher VI, VFI, PSV, and EDV were positively correlated with postoperative AMH and negatively correlated with FSH. In contrast, RI and PI were negatively correlated with AMH and positively correlated with FSH (Table 5).

**Table 5:** Correlations between preoperative ovarian vascular parameters and hormonal biomarkers at 6 months.

Preoperative parameter	r with AMH at 6 months	*P* value	r with FSH at 6 months	*P* value
VI (%)	0.398	<0.001	−0.366	<0.001
VFI (%)	0.421	<0.001	−0.391	<0.001
PSV (cm/s)	0.312	<0.001	−0.286	<0.001
EDV (cm/s)	0.371	<0.001	−0.347	<0.001
RI	−0.361	<0.001	0.382	<0.001
PI	−0.325	<0.001	0.340	<0.001

Values are Pearson correlation coefficients. AMH, anti-Müllerian hormone; FSH, follicle-stimulating hormone; VI, vascularization index; VFI, vascularization-flow index; PSV, peak systolic velocity; EDV, end-diastolic velocity; RI, resistance index; PI, pulsatility index.

The strongest positive correlation with AMH was observed for VFI (r = 0.421, *P* < 0.001), whereas RI showed the strongest positive correlation with FSH (r = 0.382, *P* < 0.001). The magnitudes of these correlations were moderate. Because these analyses were unadjusted, the findings demonstrate associations rather than independent or causal relationships between preoperative vascular characteristics and subsequent ovarian reserve biomarkers.

### 3.4 Temporal changes in ovarian reserve markers

Complete longitudinal AMH measurements were available for all 280 participants at baseline and at 3, 6, and 12 months after surgery. Accordingly, all 118 patients in the composite-outcome group and all 162 patients in the no-composite-outcome group contributed AMH measurements at each of the four prespecified time points.

Serum AMH decreased after surgery in both groups, with a more pronounced reduction among patients who met the 6-month composite outcome. In the composite-outcome group, mean AMH decreased from 2.43 ± 0.86 ng/mL preoperatively to 1.61 ± 0.65 ng/mL at 3 months and remained reduced at 6 months (1.67 ± 0.68 ng/mL), followed by partial recovery at 12 months (1.80 ± 0.72 ng/mL). In patients without the composite outcome, AMH decreased more modestly from 2.76 ± 0.89 ng/mL preoperatively to 2.31 ± 0.80 ng/mL at 3 months, followed by 2.43 ± 0.81 ng/mL at 6 months and 2.52 ± 0.83 ng/mL at 12 months (Table 6 and Figure 2).

**Table 6:** Temporal changes in serum AMH (ng/mL) after laparoscopic endometrioma cystectomy.

Time point	Composite outcome (*n* = 118)	No composite outcome (*n* = 162)	*P* value
Preoperative	2.43 ± 0.86	2.76 ± 0.89	0.002
3 months	1.61 ± 0.65	2.31 ± 0.80	<0.001
6 months	1.67 ± 0.68	2.43 ± 0.81	<0.001
12 months	1.80 ± 0.72	2.52 ± 0.83	<0.001

Data are presented as mean ± SD. Complete AMH measurements were available for all 280 participants at each time point. *P* values in the final column represent between-group comparisons at each time point. The mixed-effects model included time, group, and group-by-time interaction as fixed effects and participant as a random intercept. P_time < 0.001, P_group < 0.001, and P_group × time < 0.001. AMH, anti-Müllerian hormone.

**Figure 2:**
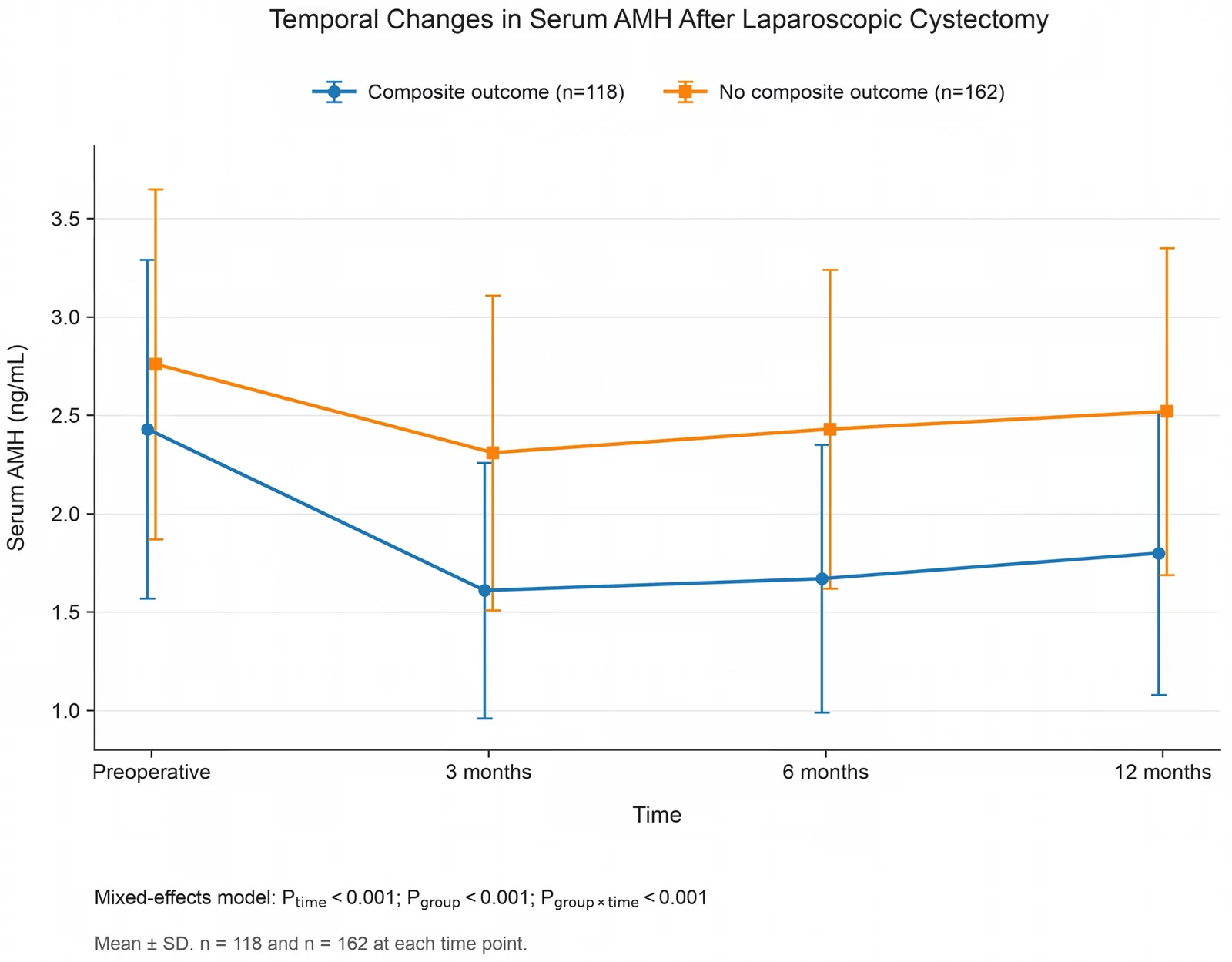
Temporal trajectories of serum anti-Müllerian hormone (AMH) before surgery and at 3, 6, and 12 months after laparoscopic endometrioma cystectomy according to the 6-month composite outcome. Data are presented as mean ± SD. Mixed-effects analysis showed significant effects of time, group, and group-by-time interaction (all *P* < 0.001).

Mixed-effects analysis demonstrated significant effects of time (*P* < 0.001), group (*P* < 0.001), and the group-by-time interaction (*P* < 0.001), indicating that postoperative AMH trajectories differed between the two groups.

### 3.5 Multivariable logistic regression analysis

Multivariable logistic regression identified age, bilateral endometriomas, maximum cyst diameter, r-AFS score, preoperative VI, and preoperative RI as factors independently associated with the 6-month composite ovarian reserve marker outcome (Table 7).

**Table 7:** Multivariable logistic regression analysis of factors associated with the 6-month composite ovarian reserve marker outcome.

Predictor	*β*	SE	OR	95% CI	*P* value
Age (per year)	0.134	0.036	1.144	1.065–1.228	<0.001
Bilateral cysts (yes vs. no)	1.160	0.363	3.190	1.566–6.500	0.001
Maximum cyst diameter (per cm)	0.377	0.113	1.458	1.167–1.821	0.001
r-AFS score (per point)	0.089	0.016	1.094	1.060–1.128	<0.001
Preoperative VI (per 1%)	−0.288	0.075	0.750	0.647–0.868	<0.001
Preoperative RI (per 0.1 unit)	1.235	0.246	3.437	2.121–5.571	<0.001

OR, odds ratio; CI, confidence interval; r-AFS, revised American Fertility Society score; VI, vascularization index; RI, resistance index.

Older age was associated with increased odds of postoperative ovarian reserve marker decline (OR = 1.144 per year, 95% CI 1.065–1.228, *P* < 0.001). Bilateral endometriomas were associated with approximately threefold higher odds than unilateral disease (OR = 3.190, 95% CI 1.566–6.500, *P* = 0.001). Larger maximum cyst diameter and higher r-AFS score were also independently associated with the outcome.

Among vascular parameters, higher VI was independently associated with lower odds of the composite outcome (OR = 0.750 per percentage-point increase, 95% CI 0.647–0.868, *P* < 0.001), whereas higher RI was associated with greater odds. For clinical interpretation, the RI effect was expressed per 0.1-unit increase; each 0.1-unit increase in RI was associated with approximately 3.44-fold higher odds of the composite outcome (OR = 3.437, 95% CI 2.121–5.571, *P* < 0.001).

### 3.6 Performance of the exploratory perioperative prediction model

The six variables retained in the multivariable analysis were combined to construct an exploratory perioperative prediction model. Using RI in its original numerical scale, the fitted probability was expressed as:

*P* = 1/[1 + exp(−*η*)]

*η* = −14.95 + 0.134  ×  Age + 1.160  ×  Bilateral cysts + 0.377 × Maximum cyst diameter + 0.089 × r-AFS−0.288 × VI + 12.35 ×  RI

Bilateral cysts were coded as 1 for bilateral disease and 0 for unilateral disease.

The composite perioperative model showed good apparent discrimination, with an AUC of 0.888 (95% CI 0.845–0.923). At the optimal probability cutoff of >0.57, sensitivity was 76.3% and specificity was 90.1%; the corresponding PPV and NPV were 84.9% and 83.9%, respectively (Table 8 and Figure 3).

**Table 8:** Discriminative performance of individual ultrasound parameters and the exploratory perioperative model.

Model/parameter	AUC (95% CI)	Cutoff	Sensitivity (%)	Specificity (%)
Preoperative VI	0.702 (0.639–0.763)	≤8.57%	75.4	58.6
Preoperative RI	0.699 (0.634–0.763)	>0.630	62.7	69.8
Composite perioperative model	0.888 (0.845–0.923)	>0.57	76.3	90.1
Bootstrap-corrected model	0.878	—	—	—

At the composite-model cutoff, TP = 90, FN = 28, TN = 146, and FP = 16, corresponding to PPV = 84.9% and NPV = 83.9%. AUC, area under the receiver operating characteristic curve; PPV, positive predictive value; NPV, negative predictive value.

**Figure 3:**
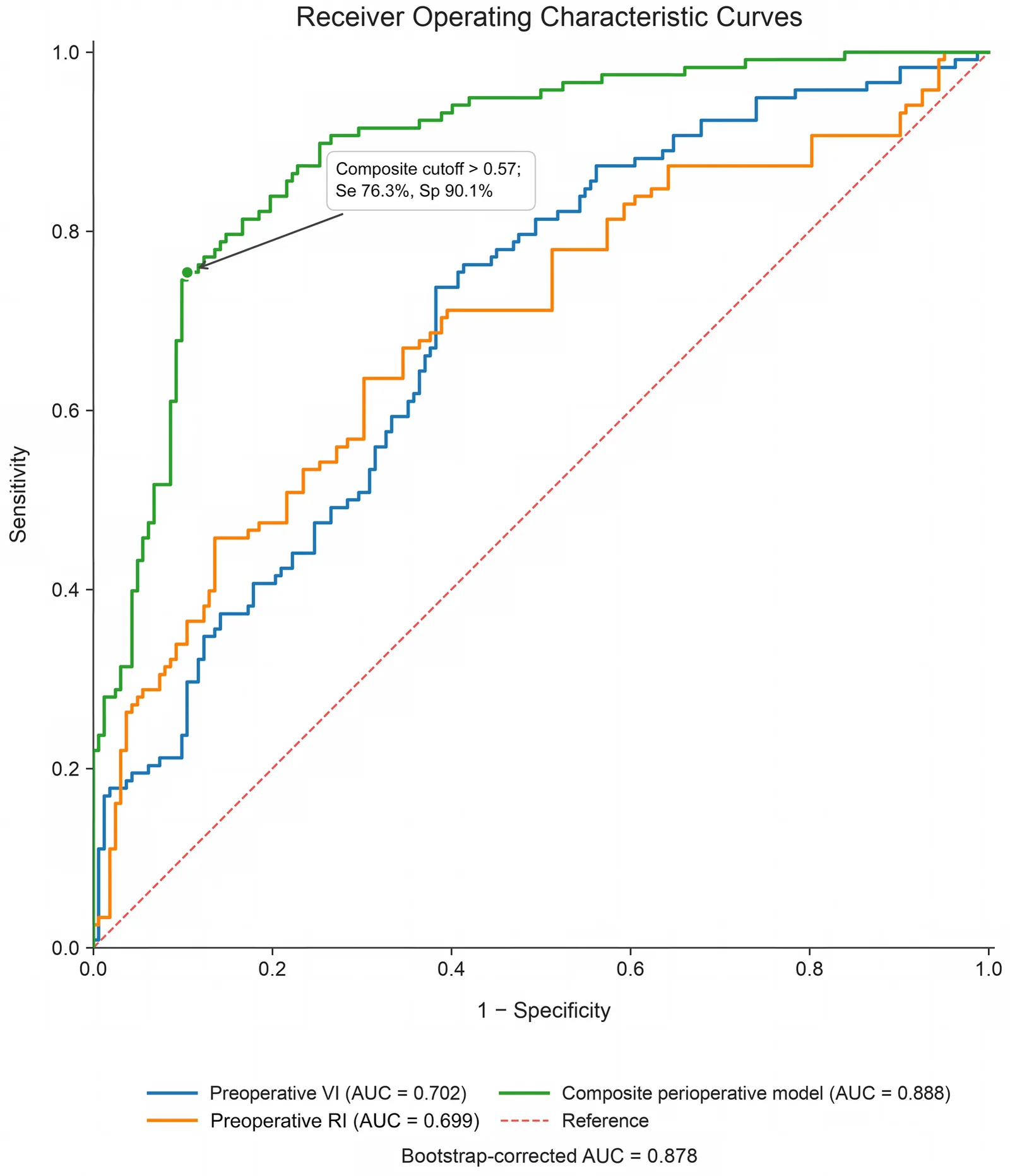
Receiver operating characteristic (ROC) curves for preoperative VI, preoperative RI, and the exploratory perioperative composite model. The apparent AUCs were 0.702 for VI, 0.699 for RI, and 0.888 for the composite model. At the probability cutoff of >0.57, the composite model yielded 76.3% sensitivity and 90.1% specificity. Bootstrap-corrected AUC was 0.878. VI, vascularization index; RI, resistance index; AUC, area under the curve.

VI and RI alone showed moderate discriminatory performance, with AUCs of 0.702 and 0.699, respectively. The combined model therefore demonstrated greater apparent discrimination than either of these individual vascular parameters considered alone.

Bootstrap internal validation yielded an optimism-corrected AUC of 0.878, indicating a modest reduction from the apparent performance. This correction reflects internal optimism within the development cohort and should not be interpreted as evidence of external validation.

### 3.7 Sensitivity analysis using VFI instead of VI

Because VFI showed a strong univariable association with the composite outcome and represents a vascular feature closely related to VI, an alternative model was fitted by replacing VI with VFI while retaining age, bilateral cysts, maximum cyst diameter, r-AFS score, and RI.

In this sensitivity model, higher VFI remained independently associated with lower odds of the composite outcome (OR = 0.483 per 1-unit increase, 95% CI 0.340–0.686, *P* < 0.001), while the effects of the remaining predictors were materially unchanged. The VFI-based model yielded an apparent AUC of 0.889 compared with 0.888 for the primary VI-based model. The corresponding bootstrap-corrected AUCs were 0.880 and 0.878, respectively (Supplementary Table S3). These findings indicate that overall model performance and the direction of associations were not materially dependent on the choice of VI versus VFI.

## 4 Discussion

This retrospective study found that preoperative ovarian vascular and hemodynamic parameters, particularly VI and RI, were associated with postoperative decline in ovarian reserve markers after laparoscopic endometrioma cystectomy. The exploratory perioperative model showed good apparent discrimination, but its performance should not be described as clinically validated because only internal bootstrap assessment was performed. External validation and comparison against models incorporating established baseline ovarian reserve markers are required.

### 4.1 Ovarian vascular and hemodynamic parameters

VI and RI were retained in the primary exploratory multivariable model. VI reflects the proportion of color-coded vascular signal within the defined 3D ovarian volume, whereas RI characterizes resistance within the sampled arterial waveform. These parameters therefore represent different but potentially complementary aspects of ovarian vascularization and hemodynamics (13, 14). The observed vascular differences may partly reflect pre-existing differences in ovarian condition, disease severity, or other measured and unmeasured factors. Importantly, baseline AMH, AFC, and ovarian volume also differed between outcome groups, indicating that some patients already had lower ovarian reserve before surgery. Therefore, the vascular parameters should not be interpreted as providing established incremental predictive value beyond conventional ovarian reserve markers without direct sequential model comparison.

Although VFI showed a strong univariable association with the composite outcome, VI and VFI capture overlapping aspects of 3D ovarian vascularization. To reduce redundancy and collinearity, VI was used as the representative 3D vascularization measure in the primary model. Replacing VI with VFI in sensitivity analysis yielded nearly identical discrimination (apparent AUC 0.889 vs. 0.888; bootstrap-corrected AUC 0.880 vs. 0.878), suggesting that the principal findings were not materially dependent on the choice of VI versus VFI.

### 4.2 Relationship with ovarian reserve markers

Preoperative VI and VFI were positively correlated with postoperative AMH and inversely correlated with FSH, whereas RI and PI showed the opposite pattern. These findings demonstrate associations between preoperative vascular/hemodynamic characteristics and subsequent ovarian reserve-related biomarkers but do not establish a causal mechanism.

Postoperative AMH and FSH are influenced by several factors, including baseline ovarian reserve, age, disease laterality, cyst burden, and surgical characteristics. The lower baseline AMH and AFC observed in patients who subsequently met the composite outcome further indicate that pre-existing ovarian reserve differences may contribute to the observed postoperative associations. Future analyses should therefore examine changes from baseline and adjust explicitly for corresponding baseline ovarian reserve markers.

### 4.3 Clinical and surgical predictors

Age, bilateral disease, cyst diameter, r-AFS score, VI, and RI were retained in the exploratory model. Age and bilateral endometrioma are clinically relevant to ovarian reserve and postoperative decline. Surgical hemostatic technique may also influence postoperative reserve: randomized evidence has compared bipolar coagulation with suturing and found differences in ovarian reserve preservation (19). Earlier clinical data also demonstrated substantial AMH reduction after laparoscopic cystectomy, particularly in bilateral endometriomas (20). Systematic review evidence has confirmed that excision of ovarian endometrioma can reduce ovarian reserve as measured by AMH (21). Because operative time and blood loss differed between outcome groups and electrocoagulation exposure was incompletely reported, surgical injury remains a potential source of confounding.

### 4.4 Prediction-model interpretation

A central unresolved question is whether vascular ultrasound provides clinically meaningful predictive information beyond established preoperative factors such as age, cyst characteristics, baseline AMH, and AFC. Although VI and RI remained independently associated with the composite outcome in the present exploratory perioperative model, the current analysis did not formally quantify incremental value over a model containing conventional ovarian reserve markers alone.

Future work should therefore compare sequential models consisting of clinical variables alone, clinical variables plus baseline AMH/AFC, and these variables plus vascular ultrasound parameters, with formal assessment of discrimination, calibration, and clinical utility. In addition, because r-AFS is determined intraoperatively, the present model should be regarded as perioperative rather than strictly preoperative. A future model intended specifically for preoperative counseling or surgical planning should exclude r-AFS and other variables unavailable before surgery.

### 4.5 Potential clinical relevance

The present findings may inform future perioperative risk-stratification research, but the current exploratory model is not ready for routine clinical use. The cutoff of 0.57 was derived in the development cohort and has not undergone external validation or decision-curve assessment. Counseling and surgical decisions should therefore continue to rely on established clinical factors, baseline ovarian reserve markers, patient reproductive goals, and guideline-based management (3, 8).

### 4.6 Limitations and future directions

Several limitations should be considered. First, the single-center retrospective design creates potential selection bias; the complete screened denominator, indication-specific surgical counts, and reason-specific exclusions could not be fully reconstructed. Second, the primary endpoint is a study-specific heterogeneous composite rather than a universally validated diminished-ovarian-reserve definition. Third, baseline ovarian reserve differed between the outcome groups, including differences in AMH, AFC, and ovarian volume. Although these baseline differences were reported, the current model did not formally quantify the incremental predictive value of vascular ultrasound beyond established ovarian reserve markers such as baseline AMH and AFC. Fourth, predictor selection incorporated clinical relevance, univariable associations, and collinearity considerations. Nevertheless, the model remains exploratory and may still be affected by model-selection uncertainty and potential overfitting. The 118 outcome events provided approximately 19.7 events per predictor for the six-variable primary model, but this does not eliminate uncertainty associated with predictor specification (22, 23). Fifth, r-AFS is intraoperative, making the current composite model perioperative rather than strictly preoperative. Sixth, bootstrap validation estimates internal optimism only; external validation is required before transportability or clinical implementation can be claimed (18, 23–25). Seventh, although longitudinal AMH changes were evaluated using a mixed-effects model with complete follow-up at the prespecified time points, other ovarian reserve-related outcomes, including AFC, FSH, LH, and E2, were not analyzed longitudinally with the same level of detail. This limits a more comprehensive assessment of postoperative ovarian reserve changes beyond AMH. Eighth, all ultrasound examinations were performed by a single sonographer, and interobserver reproducibility could not be assessed. Finally, surgical exposures such as electrocoagulation duration may confound postoperative ovarian reserve changes. Future prospective studies should follow STROBE guidance (26) and use standardized acquisition, prespecified prediction modeling, mixed-effects longitudinal analyses, calibration assessment (27), external validation, and decision-curve analysis (28).

## 5 Conclusion

Preoperative ovarian vascular and hemodynamic ultrasound parameters were associated with postoperative decline in ovarian reserve markers after laparoscopic endometrioma cystectomy. The exploratory perioperative model showed good apparent discrimination, with modest optimism estimated by bootstrap internal validation. These findings do not establish causal mechanisms or external generalizability. A strictly preoperative model that incorporates baseline AMH/AFC and excludes intraoperative variables should be developed and externally validated before clinical application.

## Data Availability

The datasets presented in this study can be found in online repositories. The names of the repository/repositories and accession number(s) can be found in the article/Supplementary Material.

## References

[1] Daniilidis A Grigoriadis G Kalaitzopoulos DR Angioni S Kalkan Ü Crestani A. Surgical management of ovarian endometrioma: impact on ovarian reserve parameters and reproductive outcomes. J Clin Med. (2023) 12(16):5324. 10.3390/jcm12165324 37629367 PMC10455819

[2] Younis JS Shapso N Ben-Sira Y Nelson SM Izhaki I. Endometrioma surgery-a systematic review and meta-analysis of the effect on antral follicle count and anti-mullerian hormone. Am J Obstet Gynecol. (2022) 226(1):33–51.e7. 10.1016/j.ajog.2021.06.102 34265271

[3] Becker CM Bokor A Heikinheimo O, ESHRE Endometriosis Guideline Group, Horne A Jansen F. ESHRE guideline: endometriosis. Hum Reprod Open. (2022) 2022(2):hoac009. 10.1093/hropen/hoac009 35350465 PMC8951218

[4] Yilmaz Hanege B Guler Cekic S Ata B. Endometrioma and ovarian reserve: effects of endometriomata per se and its surgical treatment on the ovarian reserve. Facts Views Vis Obgyn. (2019) 11(2):151–7. 31824636 PMC6897522

[5] Moreno-Sepulveda J Romeral C Nino G Perez-Benavente A. The effect of laparoscopic endometrioma surgery on anti-mullerian hormone: a systematic review of the literature and meta-analysis. JBRA Assist Reprod. (2022) 26(1):88–104. 10.5935/1518-0557.20210060 34755503 PMC8769171

[6] Baraki D Richards EG Falcone T. Treatment of endometriomas: surgical approaches and the impact on ovarian reserve, recurrence, and spontaneous pregnancy. Best Pract Res Clin Obstet Gynaecol. (2024) 92:102449. 10.1016/j.bpobgyn.2023.102449 38160479

[7] Zhang Y Zhang S Zhao Z Wang C Xu S Wang F. Impact of cystectomy versus ablation for endometrioma on ovarian reserve: a systematic review and meta-analysis. Fertil Steril. (2022) 118(6):1172–82. 10.1016/j.fertnstert.2022.08.860 36334993

[8] Riemma G De Franciscis P La Verde M Ravo M Fumiento P Fasulo DD. Impact of the hemostatic approach after laparoscopic endometrioma excision on ovarian reserve: systematic review and network meta-analysis of randomized controlled trials. Int J Gynaecol Obstet. (2023) 162(1):222–32. 10.1002/ijgo.14621 36503998

[9] Chen S Chen D Wang L Xie M. Gauze packing may be a better hemostatic method to protect ovarian reserve during laparoscopic endometrioma cystectomy than conventional hemostatic methods. Arch Gynecol Obstet. (2023) 308(3):927–34. 10.1007/s00404-023-07088-4 37330917

[10] Candiani M Ferrari SM Salmeri N Dolci C Villanacci R Bartiromo L. CO2 fiber laser vaporization for endometrioma treatment results in preserved ovarian responsiveness and improved embryo quality in infertile women undergoing ART. Minerva Obstet Gynecol. (2023) 75(4):348–56. 10.23736/S2724-606X.22.05188-0 36255166

[11] Practice Committee of the American Society for Reproductive Medicine, Penzias A Azziz R Bendikson K Falcone T Hansen K. Testing and interpreting measures of ovarian reserve: a committee opinion. Fertil Steril. (2020) 114(6):1151–7. 10.1016/j.fertnstert.2020.09.134 33280722

[12] Liu Y Pan Z Wu Y Song J Chen J. Comparison of anti-mullerian hormone and antral follicle count in the prediction of ovarian response: a systematic review and meta-analysis. J Ovarian Res. (2023) 16(1):117. 10.1186/s13048-023-01202-5 37370145 PMC10294345

[13] Choi YJ Lee HK Kim SK. Doppler ultrasound investigation of female infertility. Obstet Gynecol Sci. (2023) 66(2):58–68. 10.5468/ogs.22131 36647203 PMC10025869

[14] Wang WQ Chu GH Hou XX. A comparison of Doppler measures of ovarian blood flow between women with and without ovarian dysfunction and correlations of Doppler indices with ovarian dysfunction markers: a meta-analysis. Ann Transl Med. (2023) 11(2):110. 10.21037/atm-22-5813 36819581 PMC9929747

[15] Younis JS Shapso N Izhaki I. Is ovarian reserve reduction following endometriotic cystectomy predicted? The implication for fertility preservation counseling. Front Endocrinol (Lausanne). (2022) 13:996531. 10.3389/fendo.2022.996531 36213292 PMC9532518

[16] Cavalcante MB Sampaio OGM Câmara FEA Schneider A De Ávila BM Prosczek J. Ovarian aging in humans: potential strategies for extending reproductive lifespan. Geroscience. (2023) 45(4):2121–33. 10.1007/s11357-023-00768-8 36913129 PMC10651588

[17] Younis JS Taylor HS. The impact of ovarian endometrioma and endometriotic cystectomy on anti-mullerian hormone, and antral follicle count: a contemporary critical appraisal of systematic reviews. Front Endocrinol (Lausanne). (2024) 15:1397279. 10.3389/fendo.2024.1397279 38800489 PMC11116636

[18] Collins GS Reitsma JB Altman DG Moons KGM. Transparent reporting of a multivariable prediction model for individual prognosis or diagnosis (TRIPOD): the TRIPOD statement. Ann Intern Med. (2015) 162(1):55–63. 10.7326/M14-0697 25560714

[19] Asgari Z Rouholamin S Hosseini R Sepidarkish M Hafizi L Javaheri A. Comparing ovarian reserve after laparoscopic excision of endometriotic cysts and hemostasis achieved either by bipolar coagulation or suturing: a randomized clinical trial. Arch Gynecol Obstet. (2016) 293:1015–22. 10.1007/s00404-015-3918-4 26493551

[20] Alborzi S Keramati P Younesi M Samsami A Dadras N. The impact of laparoscopic cystectomy on ovarian reserve in patients with unilateral and bilateral endometriomas. Fertil Steril. (2014) 101(2):427–34. 10.1016/j.fertnstert.2013.10.019 24269044

[21] Raffi F Metwally M Amer S. The impact of excision of ovarian endometrioma on ovarian reserve: a systematic review and meta-analysis. J Clin Endocrinol Metab. (2012) 97(9):3146–54. 10.1210/jc.2012-1558 22723324

[22] Riley RD Snell KIE Ensor J Burke DL Harrell Jr FE Moons KGM. Minimum sample size for developing a multivariable prediction model: Part II - binary and time-to-event outcomes. Stat Med. (2019) 38(7):1276–96. 10.1002/sim.7992 30357870 PMC6519266

[23] Wolff RF Moons KGM Riley RD, PROBAST Group, Whiting PF Westwood M. PROBAST: a tool to assess the risk of bias and applicability of prediction model studies. Ann Intern Med. (2019) 170(1):51–8. 10.7326/M18-1376 30596875

[24] Iba K Shinozaki T Maruo K Noma H. Re-evaluation of the comparative effectiveness of bootstrap-based optimism correction methods in the development of multivariable clinical prediction models. BMC Med Res Methodol. (2021) 21(1):9. 10.1186/s12874-020-01201-w 33413132 PMC7789544

[25] Noma H Shinozaki T Iba K Teramukai S Furukawa TA. Confidence intervals of prediction accuracy measures for multivariable prediction models based on bootstrap-based optimism correction methods. Stat Med. (2021) 40(26):5691–701. 10.1002/sim.9148 34302372

[26] von Elm E Altman DG Egger M Pocock SJ Gøtzsche PC Vandenbroucke JP. STROBE initiative. The strengthening the reporting of observational studies in epidemiology (STROBE) statement: guidelines for reporting observational studies. Lancet. (2007) 370(9596):1453–7. 10.1016/S0140-6736(07)61602-X 18064739

[27] Van Calster B Nieboer D Vergouwe Y De Cock B Pencina MJ Steyerberg EW. A calibration hierarchy for risk models was defined: from utopia to empirical data. J Clin Epidemiol. (2016) 74:167–76. 10.1016/j.jclinepi.2015.12.005 26772608

[28] Vickers AJ Elkin EB. Decision curve analysis: a novel method for evaluating prediction models. Med Decis Making. (2006) 26(6):565–74. 10.1177/0272989X06295361 17099194 PMC2577036

